# Genomic Ultraconserved Elements Show Exceptional Association with Polycomb Proteins and Positioned Nucleosomes

**DOI:** 10.3390/biology15212035

**Published:** 2026-11-08

**Authors:** Larisa Fedorova, Yuriy L. Orlov, Oleh A. Mulyar, Alexei Fedorov

**Affiliations:** 1 CRI Genetics LLC, Santa Monica, CA 90404, USA; lvfedorova3@gmail.com (L.F.); amulyar@crigenetics.com (O.A.M.); 2 Center of Digital Health and AI in Medicine, I.M. Sechenov First Moscow State Medical University of the Ministry of Health of the Russian Federation ( Sechenov University), 119991 Moscow, Russia; orlov_yu_l@staff.sechenov.ru; 3 Agrarian and Technological Institute, Peoples’ Friendship University of Russia (RUDN University), 117198 Moscow, Russia

**Keywords:** ultraconserved elements, epigenomics, gene regulatory regions, Polycomb, ChIP-seq, chromatin structure, nucleosome positioning, human genome

## Abstract

Ultraconserved elements (UCEs) are genomic sequences exhibiting exceptional evolutionary conservation, yet their biological functions remain poorly understood. Here, we investigated protein associations with 12,813 human UCEs using large collections of publicly available ChIP-seq data. Analysis of ChIP-Atlas revealed that Polycomb-group proteins showed the strongest association with UCEs among the protein families examined. Particularly striking enrichments were observed for PHC1 (223-fold), CBX7 (130-fold), EPOP (86-fold), and PCGF2 (57-fold). Complementary analysis using the ReMap database confirmed strong UCE enrichment for PHC1, CBX7, and PCGF2, with PHC1 again showing the strongest association (212-fold). Remarkably, 43% of non-redundant PHC1 ReMap peaks overlapped UCEs. We further examined UCE positions relative to nucleosomes using NucleoATAC-defined dyads in GM12878 cells. Natural UCEs occurred significantly closer to positioned nucleosomes than expected from 1000 randomized UCE datasets, with the strongest enrichment observed within 250 bp of nucleosome dyads (2.84-fold; empirical *p* ≈ 0.001). Together, these observations reveal a strong association of UCEs with Polycomb proteins and positioned nucleosomes, suggesting that ultraconserved DNA occurs within a distinctive chromatin environment potentially related to its unusual sequence and structural properties.

## Simple Summary

Ultraconserved elements are genomic regions exhibiting exceptional (ultra) evolutionary conservation across multiple species. High-throughput sequencing and chromatin accessibility data allow us to investigate the functions of these regions in the human genome. We have analyzed the association of ultraconserved genome elements with protein-coding genes, gene regulatory regions, enhancers, and non-canonical DNA regions and revealed the strong association of these conserved regions with Polycomb proteins and positioned nucleosomes. Polycomb proteins are central regulators of developmental gene repression and epigenetic memory, thereby contributing to the establishment and maintenance of cellular identity throughout development. Polycomb proteins regulate chromatin at multiple levels, ranging from histone post-translational modifications to chromatin compaction and higher-order chromosome organization. Thus, ultraconserved DNA occurs within a distinctive chromatin environment potentially related to its unusual sequence and structural properties. The striking association between ultraconserved elements and Polycomb proteins connects two evolutionarily conserved systems that are both intimately associated with the regulation of development and cellular identity.

## 1. Introduction

The current dedUCE database contains 12,813 human ultraconserved elements (UCEs), located predominantly within intronic and intergenic regions [1]. Recent studies have demonstrated that UCEs are distributed highly non-randomly across the human genome and are particularly enriched within transcription factors and other genes with critical functions in development and embryogenesis [1,2]. The average UCE is approximately 183 nucleotides long, and UCEs are generally AT-rich but highly heterogeneous in their nucleotide sequences. Individual UCEs typically show little or no sequence similarity to one another. Nevertheless, UCEs exhibit a distinctive dinucleotide composition, including an approximately 30% enrichment of GpC dinucleotides, which may contribute to unusual DNA conformational properties [3,4]. Despite experiencing the same underlying mutational processes as the rest of the genome, UCE sequences have remained extraordinarily conserved over more than 300 million years of vertebrate evolution [1,5,6], suggesting that strong purifying selection preserves their sequences. However, the biological functions responsible for this exceptional evolutionary constraint remain poorly understood. Several functional hypotheses have been proposed [7,8,9], but none fully explains the extraordinary conservation and genomic distribution of UCEs. Here, using a bioinformatic analysis of large collections of publicly available ChIP-seq experiments, we investigated proteins preferentially associated with UCE-containing genomic regions and identified an exceptionally strong association between UCEs and selected Polycomb-group proteins.

Animal development, from early embryogenesis through the establishment of differentiated cell lineages, requires precisely coordinated transitions between distinct gene-expression programs. These transitions depend on evolutionarily conserved mechanisms that activate or repress genes at appropriate developmental stages. Polycomb-group (PcG) proteins are key components of this regulatory system, controlling developmental gene expression through chromatin modification, compaction, and higher-order chromatin organization [10]. Their functions extend beyond embryonic development: in adult tissues, Polycomb proteins contribute to the regulation of stem-cell maintenance, differentiation, and cellular identity, while disruption of Polycomb regulation is frequently associated with cancer [10,11,12]. The relationship between UCEs and Polycomb-group proteins has remained largely unexplored. To our knowledge, a systematic genome-wide analysis demonstrating their pronounced association, particularly involving PHC1, CBX7, EPOP, and PCGF2, has not previously been reported. One possible reason why this relationship has remained unrecognized is that UCEs exhibit extensive sequence diversity and generally lack common nucleotide sequence motifs. Furthermore, Polycomb recruitment is not governed by a single universal DNA-binding consensus but involves complex interactions among chromatin-associated proteins, regulatory sequences, and epigenetic features. Consequently, conventional DNA motif-based approaches may not readily reveal associations between UCEs and Polycomb proteins. Our genome-wide analysis identifies this relationship through the integration of UCE genomic coordinates with experimentally determined protein-associated chromatin regions. Although the observed enrichment may be influenced by the preferential localization of both UCEs and Polycomb proteins within developmental regulatory regions, this possibility does not diminish the significance of identifying and quantitatively characterizing their association. Rather, it raises an important mechanistic question: whether UCEs participate directly in Polycomb recruitment or whether their association with Polycomb-regulated chromatin primarily reflects shared localization within developmental regulatory regions. Distinguishing between these possibilities will require further experimental investigation.

## 2. Materials and Methods

### 2.1. ChIP-Atlas Analysis of Protein Association with Ultraconserved Elements

Protein association with human UCEs was investigated using ChIP-Atlas (https://chip-atlas.org/, accessed on 2 November 2026), a database and analysis platform integrating publicly available ChIP-seq datasets [13]. The dedUCE dataset containing 12,813 human UCEs was used as the query set. UCE coordinates were provided in BED format using the GRCh38/hg38 human genome assembly. The dedUCE database contains 12,813 ultraconserved elements (UCEs) [1]. For ChIP-Atlas analysis, we used a subset of 12,769 UCEs located on the 24 primary human chromosomes (1–22, X, and Y), excluding 44 elements mapped to non-primary reference sequences. The genomic coordinates of the 12,769 UCEs are provided in Supplementary File S2. The ChIP-Atlas Enrichment Analysis tool (https://chip-atlas.org/, accessed on 2 November 2026) was used with Homo sapiens (hg38) as the genome, “TFs and others” as the experiment type, and all available cell types included. A MACS2 peak-score threshold of 50, corresponding to the least restrictive threshold available in the analysis interface, was applied. ChIP-Atlas peak calls are generated using MACS2 (https://pypi.org/project/MACS2/, accessed on 2 November 2026), a model-based method for identification of genomic regions enriched in ChIP-seq reads [14]. The MACS2 score used by ChIP-Atlas is defined as −10 × log10(Q-value). Therefore, a threshold of 50 corresponds to a MACS2 Q-value of 10^−5^. UCE regions were compared with randomly permuted genomic regions generated by the ChIP-Atlas Enrichment Analysis procedure. For BED input, ChIP-Atlas generates the random comparison set by randomly relocating the submitted intervals in the genome. For each ChIP-seq experiment, ChIP-Atlas reports the numbers of query and comparison random regions overlapping the corresponding peak set, and fold enrichment is calculated from the ratio (UCE vs. average random positions) of these overlap frequencies. ChIP-Atlas additionally evaluates differences between query and comparison regions using a two-tailed Fisher’s exact test and applies the Benjamini–Hochberg procedure to calculate Q-values [13].

The resulting dataset contained 33,109 ChIP-seq experiments representing 1846 distinct antigen/target labels. To identify proteins showing reproducible rather than experiment-specific UCE enrichment, results were subsequently aggregated by protein. For each protein, the numbers of UCE and randomized-region overlaps were summed across all corresponding ChIP-seq experiments. The aggregate enrichment ratio was calculated as the total number of observed UCE overlaps divided by the total number of randomized-region overlaps. No continuity correction was applied to these aggregate ratios, since the denominators were nonzero for all proteins included in the analysis. For proteins exhibiting exceptionally high UCE enrichment, the numbers of overlaps with randomized genomic regions were frequently very small or zero in individual ChIP-seq experiments. Under these conditions, experiment-level observed/expected enrichment ratios may become extremely large or undefined, making their arithmetic means particularly sensitive to small denominators. To reduce this instability, we calculated protein-level aggregate enrichment ratios by pooling the observed and randomized-region overlap counts across all experiments before calculating their ratio, rather than averaging individual experiment-level ratios. Nevertheless, aggregate enrichment estimates remain sensitive to small total randomized-overlap counts and should be interpreted with appropriate caution.

Reproducibly enriched proteins were subsequently classified into broad functional groups, including the Polycomb system, sequence-specific transcription factors, transcription machinery, transcriptional coregulators, chromatin-remodeling proteins, chromatin-modifying proteins, chromatin-binding/reader proteins, chromosome-architecture/cohesin proteins, DNA-replication/repair proteins, signaling/cell-cycle regulators, ubiquitin/SUMO/proteostasis proteins, and other nuclear regulatory proteins. RNA-associated proteins were further separated into three functional categories: (i) proteins with established direct DNA/RNA dual-binding activity; (ii) RNA-binding proteins with established chromatin or transcriptional functions; and (iii) proteins primarily involved in RNA processing or RNA binding. Because ChIP-seq detects chromatin association rather than necessarily direct protein–DNA contact, these functional classifications were used for biological interpretation and do not imply direct binding of every protein to UCE DNA.

Proteins were assigned to functional groups by manual curation based on their established biological functions, using UniProt annotations (https://www.uniprot.org/, release 2026_04, accessed on 2 November 2026) and published literature. Classification was based on the predominant functional roles of the proteins rather than on automated Gene Ontology enrichment analysis. Proteins assigned to the dual DNA/RNA-binding category were selected based on published evidence of their interactions with both DNA and RNA.

### 2.2. ReMap Analysis of Protein Association with UCEs

The ReMap 2022 database of experimentally determined transcriptional-regulator binding regions (https://remap.univ-amu.fr/, accessed on 2 November 2026) was used as a complementary ChIP-seq resource to evaluate the UCE associations identified by ChIP-Atlas [15]. ReMap provides manually curated and uniformly processed DNA-binding datasets from public repositories. For the human ReMap 2022 release, sequencing reads were aligned to the GRCh38/hg38 genome assembly using Bowtie2, and binding peaks were identified using MACS2 v2.1.1.2 with a *p*-value threshold of 1 × 10^−5^ [15]. Unlike the Q-value-based MACS2 score threshold used by ChIP-Atlas, the ReMap threshold is based on the unadjusted *p*-value. The complete human ReMap 2022 atlas comprises 8103 datasets representing 1210 transcriptional regulators. Although ChIP-Atlas and ReMap share some underlying publicly available ChIP-seq datasets, they employ different data-processing and quality-control procedures, with ReMap additionally incorporating manual curation.

For the present analysis, the human GRCh38/hg38 non-redundant ReMap 2022 peak dataset (remap2022_nr_macs2_hg38_v1_0.bed) was downloaded from the ReMap database on 9 September 2026. The non-redundant catalog combines overlapping binding regions for the same transcriptional regulator across available datasets and experimental conditions, thereby providing a consolidated set of genomic binding regions for each protein.

The 50 proteins showing the highest UCE/random enrichment in the ChIP-Atlas analysis were selected for complementary evaluation with ReMap. Of these proteins, 34 were represented in the ReMap non-redundant dataset. Genomic coordinates of ReMap peaks were compared with those of the natural human UCEs and with 1000 randomized UCE datasets using custom Perl programs provided in the Supplementary File S2 PROGRAMS_DB_README. The randomized datasets were generated as described previously [2] and preserved the natural UCE length and clustering distributions while containing approximately the same number of genomic regions as the natural UCE dataset. Randomized UCE datasets were generated using Perl’s built-in rand() function without a fixed seed; the datasets used for the reported analyses are provided in Supplementary File S2 to ensure computational reproducibility.

For each protein, the number of non-redundant ReMap peaks overlapping natural UCEs was determined and compared with the mean number of overlapping peaks obtained across the 1000 randomized UCE datasets. The ratio of UCE–peak overlap events to the total number of non-redundant ReMap peaks was calculated for each protein. Because individual peaks may overlap multiple UCEs, this ratio does not necessarily represent the fraction of distinct peaks overlapping UCEs. The same overlap-counting algorithm and criteria were applied to both the natural UCE dataset and all 1000 randomized UCE datasets, ensuring that observed and expected overlap counts were directly comparable.

The standard deviation of the randomized overlap counts was also calculated. In addition, the fraction of all non-redundant ReMap peaks overlapping natural UCEs was calculated as the number of UCE-overlapping peaks divided by the total number of ReMap peaks for that protein. The ReMap enrichment ratios were used as descriptive measures of genomic association. Statistical significance was assessed using 1000 randomized UCE datasets, with one-sided empirical *p*-values calculated as (r + 1)/1001, where r is the number of randomized datasets yielding overlap counts at least as large as the observed count. The resulting *p*-values were adjusted for multiple testing across the 34 proteins using the Benjamini–Hochberg procedure. These results are present in the Supplementary Table S3.

### 2.3. Nucleosome Positioning Analysis

Nucleosome positions were obtained from the NucleoATAC dataset GSE66386 generated by Schep et al. [16] for the human GM12878 lymphoblastoid cell line. NucleoATAC infers high-resolution nucleosome positions from the characteristic ATAC-seq fragment-size and fragment-position pattern surrounding nucleosomes. The processed human nucleosome-position file GM.nucpos.bed was extracted from the supplementary archive GSE66386_all_nucpos.bed.tar.gz, downloaded from the NCBI Gene Expression Omnibus (GEO; accession GSE66386; https://www.ncbi.nlm.nih.gov/geo/query/acc.cgi?acc=GSE66386, accessed on 8 October 2026). The original human coordinates were reported on the hg19 assembly. The first three BED fields, representing chromosome and the one-base dyad interval, were used for positional analysis. Because NucleoATAC calling in this dataset was performed in highly mappable open-chromatin regions, the resulting positions represent called nucleosomes within the assayed/callable regulatory chromatin rather than an unbiased genome-wide census of all nucleosomes.

The GM12878 nucleosome dyad coordinates were converted from hg19 to GRCh38/hg38 using the UCSC liftOver program [17]. Of 67,385 input dyad intervals, 67,384 were successfully mapped to hg38. The center of each one-base BED interval, calculated as (start + end)/2, was used as the dyad coordinate. Natural UCE coordinates were taken from the hg38 dedUCE BED file [1] containing 12,769 UCEs used for ChIP-Atlas. UCE midpoints were calculated identically as (start + end)/2. Thirteen natural UCEs on chromosome Y were excluded because no corresponding nucleosome dyad calls were present, leaving 12,756 analyzable natural UCEs.

For each UCE midpoint, the nearest nucleosome dyad on the same chromosome was identified using custom Perl programs and a binary-search procedure on chromosome-specific sorted dyad coordinates. All Perl programs and supporting and README files are provided in Supplementary File S2. Absolute midpoint-to-dyad distances were calculated for the natural UCE dataset and for 1000 randomized UCE datasets. The total number of UCEs varied slightly among randomized datasets as expected from the randomization procedure [2]. Consequently, comparisons of distance categories were based on fractions of analyzable UCEs rather than raw counts.

For the broad-distance analysis, the fractions of analyzable UCEs with midpoints ≤50, ≤100, ≤250, ≤500, and ≤1000 bp from the nearest dyad were calculated. Complementary fractions located >10 kb and >25 kb from the nearest dyad were also determined. Mean and median nearest-dyad distances were calculated for each dataset. For fine-scale positional analysis, signed distances were defined as UCE midpoint minus nucleosome dyad coordinate; negative values therefore indicate a UCE midpoint upstream of the dyad and positive values indicate a downstream position. UCEs within ±250 bp of the nearest dyad were summarized in consecutive 10 bp bins. Bin frequencies were normalized to all analyzable UCEs in each dataset, rather than only to UCEs within the ±250 bp window.

Natural UCE values were compared with the empirical distributions obtained from 1000 randomized UCE datasets. For proximity measures (≤50 to ≤1000 bp), enrichment was evaluated in the direction of larger fractions for natural UCEs; for long-distance measures (>10 kb and >25 kb) and nearest-distance statistics, the corresponding direction of smaller distances or fractions was evaluated. One-tailed empirical *p* values were calculated using the +1 correction, *p* = (*r* + 1)/(*N* + 1), where r is the number of randomized datasets with a value at least as extreme as the natural observation and *N* = 1000. Thus, the minimum attainable empirical *p* value was 1/1001 = 0.000999. The Benjamini–Hochberg procedure was applied across the UCE distance categories. The 10 bp positional bins were analyzed descriptively, without assigning statistical significance to individual bins. We used modeling approaches for genome region distribution estimation from [18,19].

Natural/random enrichment ratios were calculated by dividing the natural fraction by the mean fraction across the 1000 randomized datasets. The 10 bp profile was used primarily to examine the spatial shape of the association; individual fine-scale bins were interpreted cautiously because of the limited number of natural UCEs within ±250 bp and the multiple bins examined.

## 3. Results

### 3.1. Analysis of the ChIP-Atlas Database

**Figure 1:**
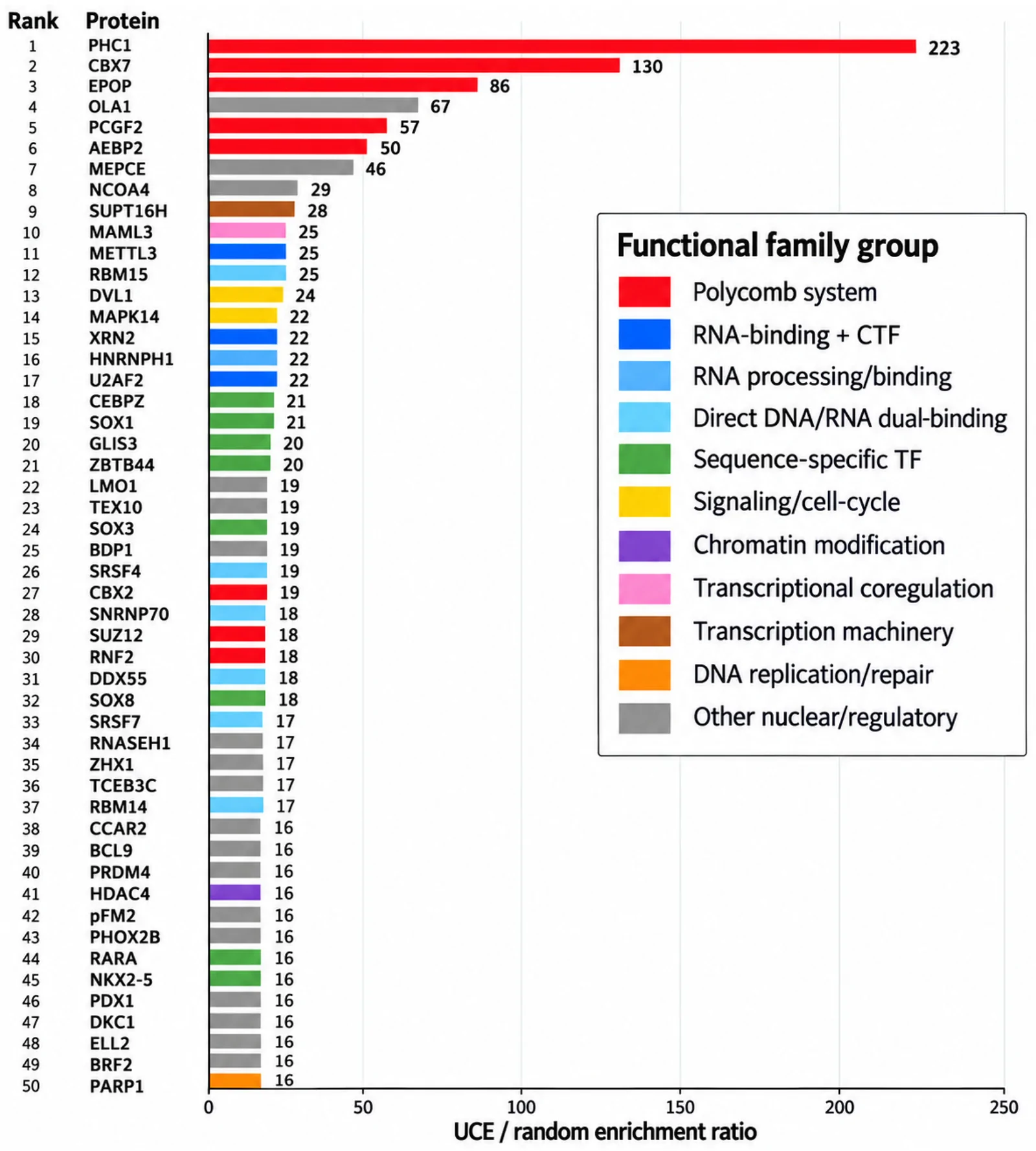
Top 50 proteins showing the best enrichment of ChIP-seq peaks at human UCEs.

Chromosomal coordinates of all 12,813 human UCEs from the dedUCE database were analyzed bioinformatically using the ChIP-Atlas public web resource to identify proteins whose ChIP-seq peaks preferentially overlap UCE sequences. This analysis, using a reproducibility requirement of ≥3 independent experiments in the database, identified 1121 proteins showing enrichment at UCEs over random expectation (Supplementary Table S1). The initial selection required an enrichment ratio greater than 1, without imposing a minimum number of cell types. For random expectation we used a built-in feature in the ChIP-Atlas resource that generates 100 randomly permuted genomic regions from our input data of 12,769 UCE chromosomal coordinates. The raw 3.1 MB output file from ChIP-Atlas is provided as Supplementary File S1, while the bioinformatically processed and sorted results are presented in Supplementary Table S1. Proteins that are associated with UCE sequences were ranked according to their enrichment, and the 50 most highly enriched proteins by their UCE associations are presented in Table 1 and visualized in Figure 1. These 50 proteins exhibited enrichment ratios ranging from 15.56 to 222.75, corresponding to more than 15-fold enrichment relative to random expectation. To assess the robustness of these findings, we repeated the ChIP-Atlas analysis using more stringent MACS2 thresholds of 100 and 200. The principal Polycomb-associated proteins retained strong enrichment at higher thresholds, although the overall protein rankings changed (Supplementary Table S2). Polycomb-system proteins were significantly overrepresented among the 50 highest-ranking proteins compared with all 1846 tested antigens from the raw ChIP-Atlas output file (8 observed versus 0.60 expected; Fisher’s exact test, *p* = 3.91 × 10^−8^).

Proteins identified by ChIP-Atlas enrichment analysis were aggregated across individual ChIP-seq experiments and classified into broad functional families. The first column “Protein” indicates the ChIP-seq antigen/target. The second column “Protein family” indicates the functional group assigned to each protein based on its established biological role. The third column “Experm” is the total number of ChIP-seq experiments available for the protein and included in the analysis. “Cell types” column indicates the number of distinct ChIP-Atlas biological/cell-type categories represented by these experiments. “Fraction enriched” is the percentage of experiments in the ChIP-Atlas database in which UCE regions showed greater overlap with ChIP-seq peaks than randomly permuted genomic regions (UCE/random overlap ratio >1). “Enrichment” is the aggregate UCE/random enrichment ratio across all individual experiments for the protein. “UCE overlaps per experm” is the median number of UCE regions overlapping ChIP-seq peaks across the individual experiments for that protein. “Median peaks per experm” indicates the median number of ChIP-seq peaks among all experiments with a particular protein. Proteins in the table are sorted by their enrichment UCE/random ratio.

A striking feature of these results was the prominent representation of Polycomb proteins among the most strongly UCE-associated proteins. Among the five most UCE-associated proteins, four belong to the Polycomb family: PHC1 (enrichment, 223-fold), CBX7 (130-fold), EPOP (86-fold), and PCGF2 (57-fold). The only non-Polycomb protein in this group, OLA1 (Obg-like ATPase 1), belongs to the OBG family and has 67-fold enrichment. Yet OLA1 has approximately 10-fold fewer UCE overlaps than the average of the above-mentioned four Polycomb proteins. Thus, OLA1 likely has limited chromosomal associations with an average of 16 UCE sites per ChIP-seq experiment. According to Table 1, each of PHC1, CBX7, and PCGF2 has more than one hundred ChIP-seq peaks that overlap with UCEs per experiment. For PHC1 and CBX7, the ratios of median UCE overlaps per experiment to median ChIP-seq peaks per experiment were approximately 44% and 31%, respectively (Table 1). These ratios provide descriptive comparisons and do not represent the fractions of distinct peaks overlapping UCEs.

Figure 1 gives a visual representation of the data from Table 1. Proteins are classified by functional family as indicated by the colors. Enrichment represents the increased overlap of UCE regions with ChIP-seq peaks relative to randomly permuted genomic regions. The prominent representation of Polycomb-system proteins (shown in red) among the most highly enriched proteins indicates a strong association between UCEs and Polycomb-associated chromatin.

Among the 37 Polycomb proteins known in humans, 22 are represented in our ChIP-Atlas dataset. We compared UCE enrichment across different functional protein families, including the Polycomb system (Table 2). The UCE/random enrichment ratio was calculated for each protein, and the mean and median enrichment values were then determined for each family. Protein families in Table 2 are ranked according to their mean enrichment. Notably, the Polycomb system showed the highest mean UCE/random enrichment among all protein families analyzed. Although the difference between Polycomb and other protein families was less pronounced when median enrichment was considered, the Polycomb median remained substantially higher than those of the other families. This indicates that the strong UCE association of the Polycomb system is not solely attributable to the exceptionally high enrichment of PHC1, CBX7, EPOP, and PCGF2; other Polycomb proteins also show a considerable preference for association with UCEs. The particularly strong UCE associations of PHC1, CBX7, EPOP, and PCGF2 are considered further in the Discussion Section.

Proteins were classified into broad functional families according to their established biological functions. For each protein, the aggregate UCE/random enrichment ratio was calculated from the cumulative numbers of UCE and randomly permuted genomic regions overlapping ChIP-seq peaks across all experiments for that protein. Column “Proteins” indicates the number of proteins assigned to each functional family. “Mean UCE/random enrichment” and “Median UCE/random enrichment” represent, respectively, the arithmetic mean and median of the protein-level aggregate enrichment ratios within each family. Protein families are ranked in descending order according to mean UCE/random enrichment.

### 3.2. Analysis of the ReMap Database

To verify the strong association of genomic UCE sequences with Polycomb proteins observed in the ChIP-Atlas analysis, we analyzed ReMap, a complementary, manually curated public resource of ChIP-seq data. Specifically, we used the non-redundant ReMap dataset, which provides the genomic locations of distinct ChIP-seq peaks identified across all available experiments for each protein (column 2 in Table 3). Because ReMap does not provide a built-in enrichment analysis based on randomized genomic regions, we used our previously generated 1000 randomized UCE datasets described in Fedorova et al. (2026) [2]. Each randomized dataset contained approximately the same number of genomic regions as the natural dedUCE dataset of 12,813 UCEs (within approximately ±100 regions) and preserved the natural UCE length and clustering distributions.

**Figure 2:**
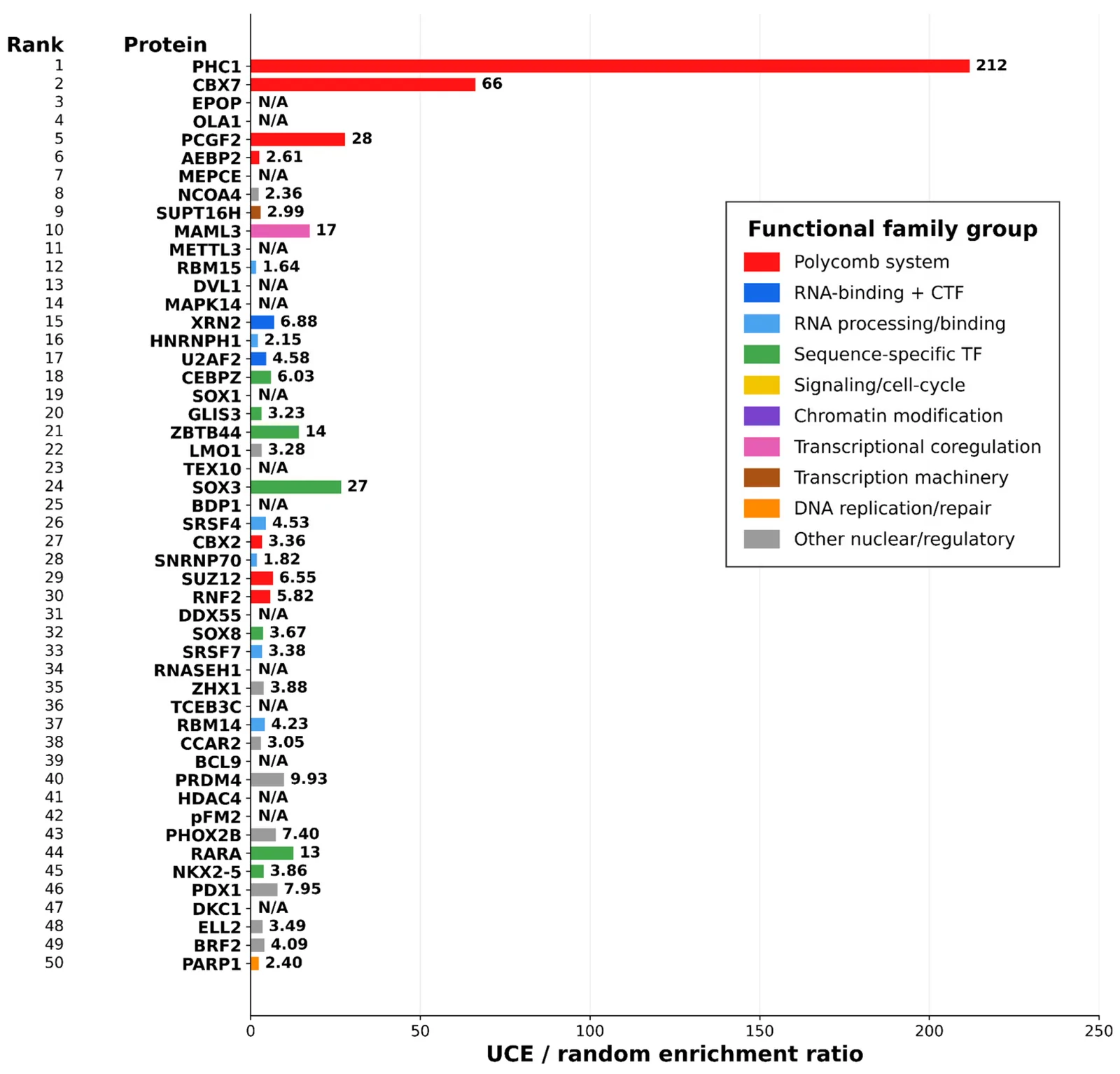
ReMap analysis of ChIP-seq peak enrichment at human UCEs.

Using our custom Perl scripts, we compared the genomic coordinates of ReMap ChIP-seq peaks with those of the natural UCEs and the 1000 randomized UCE datasets. The 50 proteins with the highest UCE enrichment identified by ChIP-Atlas (Table 1) were examined. Of these 50 proteins, 16 were not represented in the ReMap database (EPOP, OLA1, MEPCE, METTL3, DVL1, MAPK14, SOX1, TEX10, BDP1, DDX55, RNASEH1, TCEB3C, BCL9, HDAC4, pFM2, DKC1); consequently, Table 3 presents results for the remaining 34 proteins for which ReMap ChIP-seq data were available. The corresponding graphical representation is shown in Figure 2.

The ReMap analysis confirmed the strong enrichment of ChIP-seq peaks at UCEs for three of the four most highly enriched Polycomb proteins identified by ChIP-Atlas: PHC1, CBX7, and PCGF2; data for EPOP were not available in ReMap. The strongest enrichment was again observed for PHC1, with a 212-fold excess of UCE-overlapping peaks relative to random expectation. Remarkably, 185 of the 433 non-redundant PHC1 peaks (43%) overlapped at least one UCE, yielding a total of 232 UCE–peak overlap events (Table 3). In addition, among the 12,769 analyzed UCEs, 232 distinct UCEs (1.82%) overlapped PHC1 peaks under the overlap-counting criteria used in our analysis. Each of these UCEs contributed a single UCE–peak overlap event. CBX7 showed the second-highest enrichment (66-fold in ReMap compared with 130-fold in ChIP-Atlas), while PCGF2 showed a 28-fold enrichment. The enrichment data from Table 3 are presented graphically in Figure 2 for comparison with the same analysis of ChIP-Atlas (see Figure 1). Thus, two complementary ChIP-seq resources identified the same Polycomb proteins among those showing the strongest associations with human UCEs. The particularly strong associations of PHC1 and CBX7 with UCEs are considered further in the Discussion section. Statistical analysis using 1000 randomized UCE datasets demonstrated significant enrichment for 33 of the 34 proteins examined (BH-FDR < 0.05; Supplementary Table S3). The strongest Polycomb-associated proteins, PHC1, CBX7, and PCGF2, each exhibited an empirical *p*-value of 0.000999 (BH-FDR = 0.001132).

The 50 proteins showing the highest UCE enrichment in the ChIP-Atlas analysis (Table 1) were examined using the non-redundant ReMap ChIP-seq dataset; 34 of these proteins were represented in ReMap and are shown. The column “Protein” indicates the ChIP-seq target. “Peaks” is the total number of non-redundant ReMap ChIP-seq peaks available for that protein. “UCE” is the number of UCE-peak overlaps. “Random” is the mean number of peaks overlapping randomized UCE regions across 1000 randomized UCE datasets, and “SD” is the corresponding standard deviation. “Enrichment” is the ratio of the observed number of UCE-overlapping peaks to the mean number expected from the randomized datasets (UCE/Random). “Overlaps/peaks” is the number of reported UCE–peak overlap events divided by the total number of non-redundant peaks.

Differences in quality-control filters for ChIP-seq experiments and, in some cases, small numbers of peaks may explain variations between Figure 1 and Figure 2. However, the main trend with Polycomb proteins remains the same.

The 50 proteins with the highest UCE enrichment identified by the ChIP-Atlas analysis (Table 1 and Figure 1) were examined using the non-redundant ReMap ChIP-seq dataset. Enrichment represents the ratio between the observed number of ReMap ChIP-seq peaks overlapping natural UCEs and the mean number of peaks overlapping randomized UCE regions across 1000 randomized UCE datasets. Proteins are shown in the same order and with the same functional-family colors as in Figure 1. N/A indicates proteins for which ChIP-seq data were not available in ReMap. The ReMap analysis confirmed particularly strong UCE enrichment for the Polycomb proteins PHC1, CBX7, and PCGF2.

### 3.3. Analysis of UCE Positions Relative to Nucleosomes

**Figure 3:**
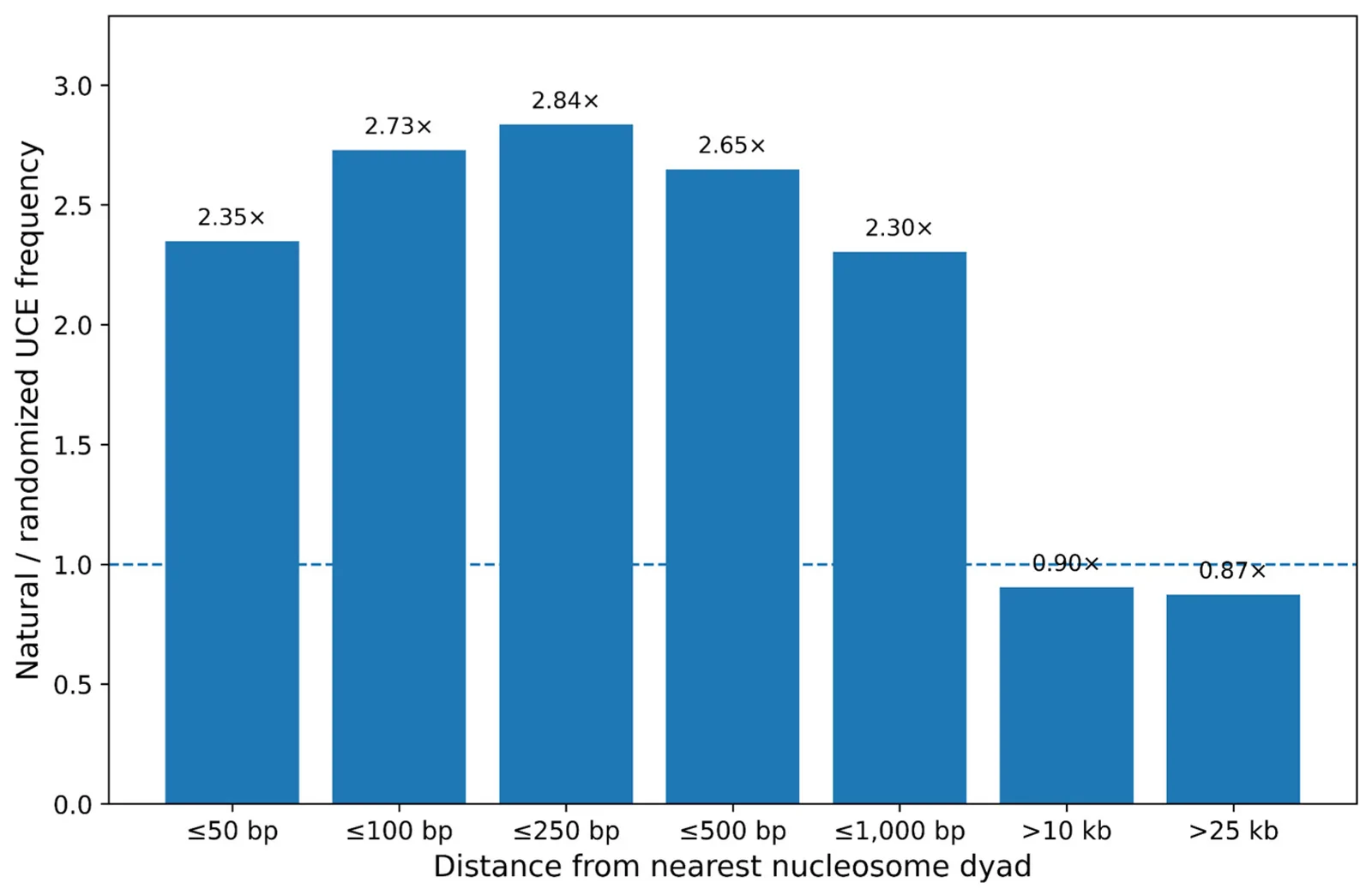
Enrichment of natural ultraconserved elements near positioned nucleosomes. Bars show the ratio between the frequency observed for natural UCEs and the mean frequency obtained from 1000 randomized UCE datasets for increasing distance thresholds from the nearest GM12878 NucleoATAC nucleosome dyad. Values >1 indicate enrichment and values <1 indicate depletion relative to randomized UCE distributions. Natural UCEs were enriched 2.35-, 2.73-, 2.84-, 2.65-, and 2.30-fold within 50, 100, 250, 500, and 1000 bp, respectively, whereas UCEs were underrepresented among loci located >10 kb and >25 kb from the nearest called dyad.

To examine whether UCEs show a non-random relationship with positioned nucleosomes, we compared the midpoints of 12,756 natural human UCEs with publicly available nucleosome dyad positions inferred by NucleoATAC in the GM12878 lymphoblastoid cell line. The dataset contained 67,385 nucleosome dyad positions within NucleoATAC callable/open-chromatin regions. Because the NucleoATAC dyads were called within selected accessible genomic regions, this dataset represents positioned nucleosomes in callable chromatin rather than a genome-wide map of nucleosome positions. We computed the distance from each UCE midpoint to the nearest nucleosome dyad. The results and corresponding statistics are provided in Table 4 and summarized in Figure 3.

Frequencies were calculated relative to the number of analyzable UCEs in each dataset. Random values are based on 1000 randomized UCE datasets. Empirical *p* values are one-tailed and use the +1 correction, *p* = (*r* + 1)/(N + 1), where r is the number of randomized datasets at least as extreme as the natural value in the tested direction and N = 1000. For all categories shown, *r* = 0 and *p* = 1/1001 = 0.000999. The Benjamini–Hochberg correction was applied across the seven distance categories. All seven BH-adjusted *p*-values were 0.000999, identical to the corresponding empirical *p*-values.

The median distance from a natural UCE midpoint to the nearest called nucleosome dyad was 24.85 kb, compared with a mean randomized median of 32.42 kb across 1000 randomized UCE datasets. The mean distance was also markedly lower for natural UCEs (88.22 kb) than for randomized UCEs (167.82 kb). Importantly, although UCEs exhibited a 2.84-fold enrichment within 250 bp of NucleoATAC-defined nucleosome dyads compared with randomized datasets, this proximity was observed for only 2.916% of UCEs, indicating that the association involves a relatively small subset rather than representing a general property of UCEs.

**Figure 4:**
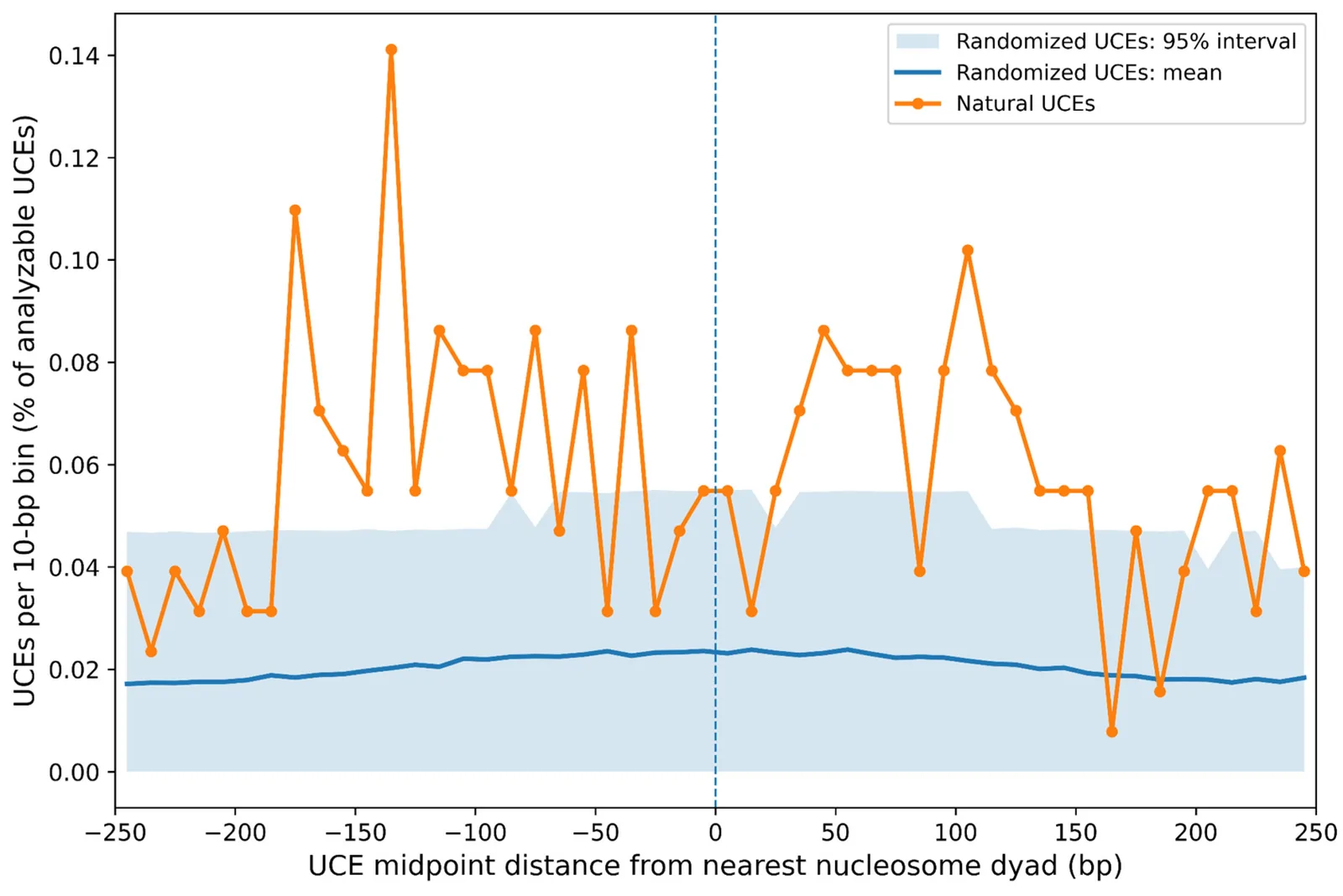
Fine-scale distribution of UCE positions relative to nucleosome dyads. Distances between UCE midpoints and the nearest GM12878 NucleoATAC nucleosome dyad were analyzed in 10 bp bins across a ±250 bp interval. The orange line represents the frequency of natural UCEs in each bin, the blue line represents the mean frequency obtained from 1000 randomized UCE datasets, and the shaded area indicates the 95% interval of the randomized distributions. Frequencies were calculated relative to all analyzable UCEs in each dataset. The vertical dashed line indicates the nucleosome dyad position (0 bp). Natural UCEs show increased frequencies at multiple positions surrounding nucleosome dyads rather than a single sharp enrichment centered at the dyad.

Figure 3 demonstrates that natural UCEs were strongly enriched near nucleosome dyads. For all distance thresholds examined, the natural UCE values were more extreme than those of all 1000 randomized datasets (one-tailed empirical *p* = 1/1001 ≈ 0.001). A fine-scale analysis using 10 bp bins across ±250 bp did not reveal a dominant sharp peak at the nucleosome dyad itself (Figure 4). Instead, natural UCEs were enriched relative to randomized expectations at multiple positions surrounding the dyad. This pattern suggests a broader local association rather than precise alignment of computationally defined UCE midpoints with nucleosome centers. Such a pattern is also consistent with the computational definition of UCE boundaries, which depends on conservation thresholds and filtering, while adjacent sequences frequently retain substantial evolutionary conservation.

These results provide independent evidence that UCEs are associated with a distinctive chromatin environment. Natural UCEs occur substantially closer to NucleoATAC-defined nucleosome dyads than expected from randomized UCE distributions, with the strongest broad-bin enrichment observed within 250 bp. The absence of a narrow central peak is not unexpected because the midpoint of a computationally defined UCE is not necessarily a biologically privileged position, and conserved sequence often extends beyond the formal UCE boundaries. Together with the strong UCE associations observed for selected Polycomb proteins, particularly PHC1 and CBX7, these results provide complementary evidence that UCEs occur in non-random chromatin environments.

## 4. Discussion

### 4.1. PHC1, CBX7, and EPOP Polycomb Proteins

Our bioinformatics investigation revealed that Polycomb-group proteins have the most pronounced association with UCEs among the 1121 proteins examined in this study. Polycomb proteins are central regulators of developmental gene repression and epigenetic memory, thereby contributing to the establishment and maintenance of cellular identity throughout development. They regulate chromatin at multiple levels, ranging from histone post-translational modifications to chromatin compaction and higher-order chromosome organization [10]. The Polycomb system is organized principally into two major classes: Polycomb Repressive Complex 1 (PRC1) and Polycomb Repressive Complex 2 (PRC2). PRC1 catalyzes monoubiquitylation of histone H2A at lysine 119 (H2AK119ub1), whereas PRC2 catalyzes mono-, di-, and trimethylation of histone H3 at lysine 27 (H3K27me1/2/3) [10,11,12]. Importantly, the UCE-associated Polycomb proteins identified in our study include components of both PRC1 and PRC2, suggesting that the observed association is not restricted to a single Polycomb complex.

Among all proteins analyzed, PHC1 (Polyhomeotic homolog 1) showed the strongest association with UCEs, with a 223-fold UCE/random enrichment in the ChIP-Atlas analysis. This exceptional enrichment was reproduced using ReMap. PHC1 is a component of canonical PRC1 but, unlike the catalytic RING proteins or the chromodomain-containing CBX proteins, it does not primarily function by depositing or recognizing the characteristic Polycomb-associated histone modifications. Instead, PHC proteins contribute to higher-order Polycomb complex organization through their conserved sterile alpha motif (SAM), which can mediate polymerization and is important for chromatin compaction and Polycomb-associated condensates [20,21]. PHC proteins also contain an adjacent FCS-type zinc-finger domain, while other domains contribute to PRC1 assembly. Thus, the exceptionally strong association of PHC1 with UCEs is particularly intriguing because it links UCEs to a Polycomb component involved in higher-order chromatin organization rather than primarily to the enzymatic deposition or recognition of histone modifications [22]. PHC1 may also have functions extending beyond conventional PRC1-mediated repression. For example, PHC1 has been implicated in pluripotency maintenance through PRC1-independent activation of NANOG [23]. Together, these observations raise the possibility that the unusually strong association between PHC1 and UCEs reflects a relationship between ultraconserved DNA and higher-order chromatin organization at developmentally regulated genomic loci.

The second strongest association with UCEs was observed for CBX7 (Chromobox protein homolog 7). CBX proteins, together with PHC proteins, are characteristic components of canonical PRC1 complexes. Through their chromodomains, CBX proteins recognize H3K27me3 deposited by PRC2 and thereby contribute to the recruitment and organization of canonical PRC1 at Polycomb-regulated chromatin [24]. Interestingly, CBX7 differs substantially from CBX2 in its intrinsic chromatin-compaction and phase-separation properties, which are particularly pronounced for CBX2 [25,26]. Nevertheless, CBX2 was also strongly associated with UCEs in our analysis, ranking 27th in Table 1. Like PHC1, CBX7 has important functions in pluripotent and multipotent cells, including embryonic and hematopoietic stem cells [27,28,29,30]. CBX7 has also been implicated in the regulation of pluripotency and NANOG-associated transcriptional programs [31]. Thus, the two Polycomb proteins showing the strongest UCE associations, PHC1 and CBX7, converge functionally on chromatin regulation in stem and developmental contexts.

Among PRC2-associated proteins, EPOP (Elongin BC and Polycomb Repressive Complex 2-associated protein) showed the highest UCE/random enrichment. EPOP is a PRC2-associated scaffolding protein whose regulatory functions remain incompletely understood [32]. Recent evidence indicates that EPOP can promote PRC2 chromatin association in a DNA-sequence-dependent manner [33,34]. EPOP also recruits the Elongin BC complex to chromatin in pluripotent stem cells [35,36]. These properties make the strong association of EPOP with UCEs particularly interesting, although its absence from the ReMap dataset prevented complementary confirmation in our second analysis.

### 4.2. How Polycomb Proteins May Interact with UCE Genomic Sequences

Despite extensive investigation of mammalian Polycomb proteins, the mechanisms that determine their recruitment to particular genomic loci remain incompletely understood. Unlike many sequence-specific transcription factors, Polycomb complexes do not recognize a single consensus DNA motif that can account for their genomic distribution. CpG-rich DNA and unmethylated CpG islands have been implicated in Polycomb recruitment, together with multiple chromatin-associated proteins and epigenetic features, suggesting that recruitment is likely determined by several complementary mechanisms [33].

Although many UCEs have been proposed to function as developmental enhancers, enhancer activity alone does not readily explain their extraordinary sequence constraint [7]. UCEs frequently extend for more than 200 bp, with extreme conservation distributed across most or all of the nucleotides rather than being restricted to short recognizable regulatory motifs. Moreover, individual UCEs are highly heterogeneous in primary sequence and lack a common sequence motif shared by a substantial fraction of elements. Previously, we demonstrated that UCEs have an unusual dinucleotide composition, including significant enrichment of GpC dinucleotides [6], and proposed that these sequences may adopt distinctive DNA conformational properties [3,4]. The present results raise an intriguing extension of this hypothesis. PHC1 contains an FCS-type zinc-finger domain whose functional contribution to DNA recognition remains incompletely characterized. We therefore propose that the exceptional association of PHC1 with UCEs might involve recognition, directly or indirectly, of structural features generated by the unusual sequence composition of UCE DNA. This model does not require a common primary-sequence motif among UCEs and could therefore provide a potential explanation for the association of Polycomb proteins with a highly sequence-heterogeneous class of ultraconserved elements. Because Polycomb regulation is particularly important during early development, a functional relationship between UCEs and Polycomb-regulated chromatin could also help explain the exceptionally strong purifying selection acting on these sequences. Mutations affecting functionally important UCEs could be selected against during early embryogenesis, potentially contributing to their extraordinary evolutionary conservation. The reported involvement of UCEs in cancer [9,37] provides an additional context in which disruption of UCE-associated chromatin regulation may be biologically important. However, this hypothesis remains speculative. Our computational analyses establish genomic colocalization but do not demonstrate direct recruitment of Polycomb complexes by UCEs or identify the molecular mechanisms responsible for this association. Alternative explanations, including preferential localization of both UCEs and Polycomb proteins within developmental regulatory regions, cannot be excluded. Experimental studies will be required to determine whether distinctive UCE DNA conformations contribute directly to Polycomb recruitment or whether the observed association reflects broader features of chromatin organization.

An additional limitation of our analysis is the heterogeneity of publicly available ChIP-seq datasets, which represent different cell types, biological conditions, and experimental procedures. Consequently, the observed aggregate enrichments should be interpreted as associations across the analyzed datasets rather than as evidence that these relationships are uniformly maintained across all cell types or developmental stages.

Our nucleosome analysis provides additional evidence that UCEs are associated with a non-random chromatin environment [38]. Natural UCEs were significantly enriched in proximity to positioned nucleosomes compared with 1000 randomized UCE datasets, with the strongest enrichment observed within 250 bp of NucleoATAC-defined nucleosome dyads. Although these observations do not establish a mechanistic relationship between UCE sequence properties, nucleosome positioning, and Polycomb recruitment, they are consistent with the possibility that distinctive structural properties of UCE DNA contribute to their chromatin environment.

## 5. Conclusions

Polycomb-group proteins showed the strongest association with UCEs in ChIP-Atlas data. Natural UCEs occurred significantly closer to positioned nucleosomes than expected by chance. Thus, a strong association of UCEs with Polycomb proteins and positioned nucleosomes allows us to conclude that ultra-conserved DNA occurs within a distinctive chromatin environment.

## Figures and Tables

**Table 1:** Top 50 proteins showing enrichment of ChIP-seq peaks at human UCEs.

Protein	Protein Family	Experm	Cell Types	Fraction Enriched	Enrichment	UCE Overlaps per Experm	Median Peaks per Experm
PHC1	Polycomb system	6	1	100%	223	137	312
CBX7	Polycomb system	20	5	100%	130	297	963
EPOP	Polycomb system	5	1	100%	86	50	311
OLA1	Other nuclear/regulatory	4	1	50%	67	16	129
PCGF2	Polycomb system	19	7	100%	57	102	1011
AEBP2	Polycomb system	8	2	100%	50	13	387
MEPCE	Other nuclear/regulatory	3	2	67%	46	3	37
NCOA4	Other nuclear/regulatory	4	2	100%	29	57	1027
SUPT16H	Transcription machinery	3	3	100%	28	40	971
MAML3	Transcriptional coregulation	4	1	100%	25	190	3481
METTL3	RNA-binding + CTF	9	3	100%	25	33	560
RBM15	RNA processing/binding	10	1	100%	25	35	1025
DVL1	Signaling/cell-cycle	3	1	100%	24	353	6080
MAPK14	Signaling/cell-cycle	4	1	100%	22	40	910
XRN2	RNA-binding + CTF	16	3	100%	22	58	980
HNRNPH1	RNA processing/binding	6	2	100%	22	30	728
U2AF2	RNA-binding + CTF	7	3	100%	22	12	736
CEBPZ	Sequence-specific TF	5	3	100%	21	29	1008
SOX1	Sequence-specific TF	7	2	100%	21	15	942
GLIS3	Sequence-specific TF	3	2	100%	20	56	1490
ZBTB44	Sequence-specific TF	5	3	80%	20	43	3087
LMO1	Other nuclear/regulatory	4	2	75%	19	23	685
TEX10	Other nuclear/regulatory	14	2	100%	19	3	165
SOX3	Sequence-specific TF	3	1	100%	19	2	135
BDP1	Other nuclear/regulatory	12	5	100%	19	28	1267
SRSF4	RNA processing/binding	4	2	100%	19	35	1043
CBX2	Polycomb system	13	5	85%	19	2	419
SNRNP70	RNA processing/binding	6	2	100%	18	16	313
SUZ12	Polycomb system	192	35	92%	18	41	1299
RNF2	Polycomb system	196	36	89%	18	81	1442
DDX55	RNA processing/binding	4	1	100%	18	38	972
SOX8	Sequence-specific TF	3	1	100%	18	66	2308
SRSF7	RNA processing/binding	4	2	100%	17	5	341
RNASEH1	Other nuclear/regulatory	4	1	100%	17	38	942
ZHX1	Other nuclear/regulatory	4	2	100%	17	17	903
TCEB3C	Other nuclear/regulatory	8	1	100%	17	28	1527
RBM14	RNA processing/binding	4	2	75%	17	5	405
CCAR2	Other nuclear/regulatory	6	2	100%	16	17	974
BCL9	Other nuclear/regulatory	5	1	100%	16	40	1681
PRDM4	Other nuclear/regulatory	8	4	88%	16	32	936
HDAC4	Chromatin modification	7	3	100%	16	88	3152
pFM2	Other nuclear/regulatory	4	2	100%	16	18	619
PHOX2B	Other nuclear/regulatory	16	4	100%	16	174	2875
RARA	Sequence-specific TF	60	17	97%	16	98	3464
NKX2-5	Sequence-specific TF	10	3	100%	16	36	3594
PDX1	Other nuclear/regulatory	8	3	100%	16	271	10,483
DKC1	Other nuclear/regulatory	6	2	100%	16	25	1466
ELL2	Other nuclear/regulatory	20	6	100%	16	46	1789
BRF2	Other nuclear/regulatory	4	3	100%	16	13	757
PARP1	DNA replication/repair	6	4	83%	16	13	1414

**Table 2:** UCE enrichment among functional families of ChIP-Atlas proteins.

Protein Family	Proteins	Mean UCE/Random Enrichment	Median UCE/Random Enrichment
Polycomb system	22	32.12	12.85
RNA-binding + established chromatin/transcription function	10	12.01	9.34
Primarily RNA processing/binding	35	8.91	6.75
Transcription machinery	13	8.22	6.88
Signaling/cell-cycle	15	7.56	5.27
DNA replication/repair	19	7.19	6.37
Transcriptional coregulation	14	7.09	5.44
Direct DNA/RNA dual-binding	10	6.62	6.38
Other nuclear/regulatory	556	6.25	5.34
Chromatin remodeling	21	5.87	5.24
Chromatin modification	35	5.71	5.00
Sequence-specific TF	335	5.60	4.95
Ubiquitin/SUMO/proteostasis	14	4.89	4.32
Chromatin binding/readers	11	4.78	5.05
Chromosome architecture/cohesin	11	3.28	2.60
Polycomb system	22	32.12	12.85
RNA-binding + established chromatin/transcription function	10	12.01	9.34
Primarily RNA processing/binding	35	8.91	6.75
Transcription machinery	13	8.22	6.88
Signaling/cell-cycle	15	7.56	5.27
DNA replication/repair	19	7.19	6.37
Transcriptional coregulation	14	7.09	5.44
Direct DNA/RNA dual-binding	10	6.62	6.38
Other nuclear/regulatory	556	6.25	5.34
Chromatin remodeling	21	5.87	5.24
Chromatin modification	35	5.71	5.00
Sequence-specific TF	335	5.60	4.95
Ubiquitin/SUMO/proteostasis	14	4.89	4.32
Chromatin binding/readers	11	4.78	5.05
Chromosome architecture/cohesin	11	3.28	2.60

**Table 3:** Analysis of UCE enrichment among ChIP-seq peaks using the ReMap database.

Proteins	Peaks	UCE	Random	SD	Enrichment	Overlaps/ Peaks
PHC1	433	232	1.10	1.15	211.87	0.54
CBX7	8383	1221	18.43	4.92	66.26	0.15
PCGF2	12,644	957	34.33	6.96	27.88	0.08
AEBP2	638	6	2.30	1.17	2.61	0.01
NCOA4	2234	11	4.67	2.27	2.36	0.00
SUPT16H	22,790	185	61.92	9.50	2.99	0.01
MAML3	9307	379	21.76	4.88	17.42	0.04
RBM15	113	2	1.22	0.56	1.64	0.02
XRN2	11,625	203	29.50	6.18	6.88	0.02
HNRNPH1	68,142	245	114.18	12.33	2.15	0.00
U2AF2	13,935	118	25.77	5.44	4.58	0.01
CEBPZ	2961	28	4.65	2.30	6.03	0.01
GLIS3	55,260	523	162.00	15.20	3.23	0.01
ZBTB44	34,497	1024	71.72	8.91	14.28	0.03
LMO1	43,560	313	95.51	9.79	3.28	0.01
SOX3	3264	176	6.61	2.87	26.63	0.05
SRSF4	13,389	135	29.81	6.63	4.53	0.01
CBX2	52,064	463	137.76	13.75	3.36	0.01
SNRNP70	5476	20	10.99	3.62	1.82	0.00
SUZ12	149,789	2188	333.81	23.15	6.55	0.01
RNF2	194,467	2655	456.02	27.00	5.82	0.01
SOX8	82,079	559	152.48	14.15	3.67	0.01
SRSF7	19,772	167	49.43	7.31	3.38	0.01
ZHX1	62,731	393	101.17	11.40	3.88	0.01
RBM14	8390	76	17.97	4.14	4.23	0.01
CCAR2	35,875	193	63.22	8.74	3.05	0.01
PRDM4	23,009	640	64.46	7.96	9.93	0.03
PHOX2B	112,700	1808	244.40	16.27	7.40	0.02
RARA	48,198	1414	112.00	11.80	12.63	0.03
NKX2-5	20,760	155	40.19	6.54	3.86	0.01
PDX1	138,396	1900	238.89	17.77	7.95	0.01
ELL2	70,789	453	129.82	13.00	3.49	0.01
BRF2	2799	19	4.64	1.98	4.09	0.01
PARP1	28,074	168	69.87	8.35	2.40	0.01

**Table 4:** Association of natural UCEs with NucleoATAC nucleosome dyads.

Distance	Natural UCEs (%)	Random Mean (%)	Random SD (%)	Natural/Random	Empirical *p*
≤50 bp	0.549	0.234	0.044	2.35	0.0010
≤100 bp	1.254	0.460	0.062	2.73	0.0010
≤250 bp	2.916	1.029	0.098	2.84	0.0010
≤500 bp	4.782	1.806	0.136	2.65	0.0010
≤1000 bp	7.306	3.172	0.189	2.30	0.0010
>10 kb	70.328	77.796	0.578	0.90	0.0010
>25 kb	49.828	57.073	0.826	0.87	0.0010

## Data Availability

The original data presented in the study are openly available in the Supplement to this manuscript (File S1, Table S1) and online at https://fedorovlab.com/UCE6/ (1 November 2026).

## Supplementary Materials

The following supporting information can be downloaded at: https://www.mdpi.com/article/10.3390/biology15212035/s1, Supplementary File S1 contains the original, unprocessed ChIP-Atlas output (wabi_chipatlas_2026-0909-1945-44-928-968409). Supplementary Table S1 contains the processed and organized results derived from ChIP-Atlas unprocessed output. Supplementary Table S2 compares ChIP-Atlas enrichment results at MACS2 peak-score thresholds of 50, 100, and 200. Supplementary Table S3 presents statistical analysis of associations between ultraconserved elements (UCEs) and protein-binding regions identified in the ReMap database. Supplementary File S2 contains databases, all Perl programs, and instructions for them (PROGRAMS_DB_README).

## Abbreviations

The following abbreviations are used in this manuscript:

UCE	Ultraconserved Elements
ChIP-seq	Chromatin immunoprecipitation followed by sequencings
