# Supplementary material for: Genomic Ultraconserved Elements Show Exceptional Association with Polycomb Proteins and Positioned Nucleosomes
Source: Biology (Basel). 2026 Nov 8;15(21):2035. doi: 10.3390/biology15212035 (PMC13761666; doi:10.3390/biology15212035)
Supplement: Supplementary file 1 [file biology-15-02035-s001.zip › SupplFileS2.tar!/PROGRAMS_DB_README/UCE6_Programs_README_r1.docx]

# **UCE6 Programs & README**

## **1. README for ReMap Processing**

### **Overview**

The Perl program process_randUCE_ChIP_v2.pl analyzes overlaps between ChIP-seq peaks and natural ultraconserved elements (UCEs), as well as 1,000 randomized UCE datasets, for 50 selected proteins from the ReMap database.

### **Running the Program**

To execute the main program in the background, use the following command:

nohup perl process_randUCE_ChIP_v2.pl &

The main program performs the following operations:

1. **Execution of Perl programs.** It initiates two Perl programs: ChIP_UCE.pl, which calculates overlaps between ReMap ChIP-seq peaks and natural UCEs, and ChIP_randUCE.pl, which processes 1,000 randomized UCE datasets.
2. **Statistical calculations.** After completing the overlap analyses, the main program calculates the mean and standard deviation of the overlap counts obtained from the 1,000 randomized UCE datasets.
3. **Protein enrichment analysis.** The program calculates UCE enrichment for 50 selected proteins, whose names must be provided in the input file prot50. Because the analysis involves 50 proteins and 1,000 randomized datasets for each protein, execution may require several hours. We therefore recommend running the program in the background and monitoring its progress by examining the nohup.out file.
4. **Output files.** Before execution, the user must create a subdirectory named RESULTS in the current working directory. All results will be written to this directory. Results for natural UCEs are stored in files named ProtName_Real (e.g., CBX7_Real). Results for individual randomized UCE datasets are stored in files named ProtName_Rand_N, where N identifies the randomized dataset (e.g., CBX7_Rand_234).
5. **Randomized UCE datasets.** The program ChIP_randUCE.pl requires 1,000 randomized UCE datasets in BED format. These files must be available in the directory RAND_UCE_1000, provided in the compressed archive RAND_UCE_1000.tar.gz.
6. **ReMap protein datasets.** The programs access the directory PROTEINS50, containing 50 files with genomic ChIP-seq peak coordinates extracted from the ReMap database. Each file is named after the corresponding protein (e.g., AEBP2, CBX2, or DDX55). These files are provided in the compressed archive PROTEINS50.tar.gz.

### **List of Supporting Programs and Files**

1. process_randUCE_ChIP_v2.pl — main Perl program coordinating the analysis and statistical calculations.
2. ChIP_randUCE.pl — Perl program analyzing randomized UCE datasets.
3. ChIP_UCE.pl — Perl program analyzing natural UCEs.
4. prot50 — text file containing the names of the 50 selected proteins.
5. RAND_UCE_1000.tar.gz — compressed archive containing 1,000 randomized UCE datasets. Should be unpacked and put in a specified folder
6. PROTEINS50.tar.gz — compressed archive containing ReMap ChIP-seq peak data for 50 proteins. Should be unpacked and put in a specified folder

## **2. README for Nucleosome Processing**

### **2.1. Perl Program UCE_nucleosome_RANDOM1000.pl**

**Purpose**

This program analyzes distances between UCE midpoints and the nearest nucleosome dyad positions identified by NucleoATAC in the human GM12878 cell line.

**Input datasets**

1. Natural UCE coordinates: UCEmattickBED.txt dedUCE database.
2. Randomized UCE datasets: RANDOM_UCE/randUCE_1 through RANDOM_UCE/randUCE_1000.
3. Nucleosome dyad coordinates: GM_dyads_hg38.bed.

**Output file**

UCE_nucleosome_random1000_summary.tsv

The program calculates distances between UCE midpoints and their nearest nucleosome dyads for the natural and 1,000 randomized UCE datasets.

For comparisons among datasets, fractions are calculated relative to the number of analyzable UCEs, defined as UCEs located on chromosomes for which nucleosome dyad data are available.

### **2.2. Perl Program UCE_nucleosome_10bp_bins.pl**

**Purpose**

This program performs a fine-scale positional analysis of UCE midpoints relative to the nearest NucleoATAC nucleosome dyad in GM12878 cells.

The analysis uses the following parameters:

- **Window:** −250 to +250 bp relative to the nearest nucleosome dyad.
- **Bin width:** 10 bp.

**Input datasets**

1. Natural UCE coordinates: UCEmattickBED.txt.
2. Randomized UCE datasets: RANDOM_UCE/randUCE_1 through RANDOM_UCE/randUCE_1000.
3. Nucleosome dyad coordinates: GM_dyads_hg38.bed.

**Output file**

UCE_nucleosome_10bp_bins.tsv

Each row of the output table represents one UCE dataset. Each positional bin contains the fraction of all analyzable UCEs whose midpoints fall within the corresponding signed-distance interval relative to the nearest nucleosome dyad.

Fractions are normalized to all analyzable UCEs in each dataset, rather than only to UCEs located within the ±250-bp window.

### **List of Supporting Programs and Files**

1. UCE_nucleosome_RANDOM1000.pl — Perl program analyzing distances between UCE midpoints and nucleosome dyads for natural and randomized UCE datasets.
2. UCE_nucleosome_10bp_bins.pl — Perl program performing fine-scale positional analysis using 10-bp bins.

**Required input datasets:**

- UCE12769.txt — natural UCE coordinates dedUCE database.
- RANDOM_UCE — directory containing 1,000 randomized UCE datasets.
- GM_dyads_hg38.bed — nucleosome dyad coordinates in the GRCh38/hg38 assembly.

**ORIGINAL dedUCE DATABASE:** Cummins, M.; Watson, C.; Edvards, R.J.; Mattick, J.S. The evolution of ultraconserved elements in vertebrates. *Mol. Biol. Evol.* **2024**, *41*, msae146.

1) merged_cumulative_97_50perc.bed
2) merged_cumulative_97_50perc.fa
